# A study on the immediate effects of enhanced external counterpulsation on physiological coupling

**DOI:** 10.3389/fnins.2023.1197598

**Published:** 2023-06-07

**Authors:** Hongyun Liu, Hui Liang, Xiaohua Yu, Yi Han, Guojing Wang, Muyang Yan, Weidong Wang, Shijun Li

**Affiliations:** ^1^Research Center for Biomedical Engineering, Medical Innovation Research Division, Chinese PLA General Hospital, Beijing, China; ^2^Key Laboratory of Biomedical Engineering and Translational Medicine, Ministry of Industry and Information Technology, Beijing, China; ^3^Department of Hyperbaric Oxygen, First Medical Center, Chinese PLA General Hospital, Beijing, China; ^4^Department of Diagnostic Radiology, First Medical Center, Chinese PLA General Hospital, Beijing, China

**Keywords:** enhanced external counterpulsation, physiological coupling, immediate effects, hemodynamics, phase synchronization

## Abstract

**Introduction:**

Enhanced external counterpulsation (EECP) is a non-invasive assisted circulation technique for its clinical application in the rehabilitation and management of ischemic cardiovascular and cerebrovascular diseases, which has complex physiological and hemodynamic effects. However, the effects of EECP on the coupling of physiological systems are still unclear. We aimed to investigate the immediate effects of EECP on the coupling between integrated physiological systems such as cardiorespiratory and cardiovascular systems.

**Methods:**

Based on a random sham-controlled design, simultaneous electrocardiography, photoplethysmography, bio-electrical impedance, and continuous hemodynamic data were recorded before, during and after two consecutive 30 min EECP in 41 healthy adults. Physiological coupling strength quantified by phase synchronization indexes (PSI), hemodynamic measurements and heart rate variability indices of 22 subjects (female/male: 10/12; age: 22.6 ± 2.1 years) receiving active EECP were calculated and compared with those of 19 sham control subjects (female/male: 7/12; age: 23.6 ± 2.5 years).

**Results:**

Immediately after the two consecutive EECP interventions, the physiological coupling between respiratory and cardiovascular systems PSI_RES–PTT_ (0.34 ± 0.14 vs. 0.49 ± 0.17, *P* = 0.002), the physiological coupling between cardiac and cardiovascular systems PSI_IBI–PTT_ (0.41 ± 0.14 vs. 0.52 ± 0.16, *P* = 0.006) and the total physiological coupling PSI_total_ (1.21 ± 0.35 vs. 1.57 ± 0.49, *P* = 0.005) in the EECP group were significantly lower than those before the EECP intervention, while the physiological coupling indexes in the control group did not change significantly (*P* > 0.05).

**Conclusion:**

Our study provides evidence that the PSI is altered by immediate EECP intervention. We speculate that the reduced PSI induced by EECP may be a marker of disturbed physiological coupling. This study provides a new method for exploring the mechanism of EECP action and may help to further optimize the EECP technique.

## 1. Introduction

Enhanced external counterpulsation (EECP) is a non-invasive auxiliary circulation technique used for the rehabilitation of cardiovascular and cerebrovascular diseases (Lin et al., 2020). Under the synchronous trigger of the electrocardiograph (ECG) R wave, the EECP device successively inflates a set of inflatable cuffs (from bottom to top) covering the patient’s lower leg, thigh, and hip. Inflation of the cuffs during diastole squeezes the arterial system of the lower part of the body, driving the blood flow back to the upper part of the body and improving blood perfusion of the heart, brain, and other important organs. The volume of blood returned to the heart is increased, while stroke volume and cardiac output are also enhanced. During the systolic period of the heart, the three-stage cuffs deflated at the same time, reducing the resistance load for cardiac blood ejection (Tian et al., 2021; Caceres et al., 2021). At present, the guidelines for the diagnosis and treatment of ischemia and stroke, stable and unstable angina, acute myocardial infarction, cardiogenic shock, congestive heart failure, and stable coronary heart disease have all included EECP therapy, in this country as well as in others.

So far, research into the effects of EECP has focused mainly on hemodynamics. The immediate hemodynamic effects, such as increased blood perfusion of tissues and organs and increased cardiac output, are of great significance for rehabilitation in patients with cardiovascular and cerebrovascular diseases. These effects are also an important basis for evaluating the clinical efficacy of EECP and optimizing therapeutic outcomes (Michaels et al., 2002; Raza et al., 2017; Lin et al., 2020). Mechanistically, EECP increases shear stress on the inner wall of blood vessels by increasing blood flow, inducing changes in gene expression in vascular endothelial cells (Raza et al., 2017). For example, the levels of tumor necrosis factor-α and other inflammatory cytokines are reduced. As a result, EECP plays a role in protecting the vascular intima and improving endothelial function, which may account for its long-term hemodynamic effects in clinical treatment (Zhang et al., 2007). In addition, while EECP may not change the characteristic parameters of heart rate variability (HRV) that reflect cardiac function during the treatment of ischemic heart disease and angina pectoris (Michaels et al., 2007; Akbarzadeh et al., 2011; Liu et al., 2023), it can have a significant immediate effect on blood pressure. Moreover, EECP may also exert time-cumulative effects, mainly manifesting as reductions in systolic blood pressure (Campbell et al., 2008; Beck et al., 2015; Liang et al., 2021). To date, it appears that there has been no research into the immediate effects of EECP on integrated physiological systems such as the cardiorespiratory and cardiovascular systems.

The human body is a complex system, and its tissues, organs, and systems exhibit different degrees of interaction or coupling at different spatial and time scales. Such interactions are crucial for the maintenance of homeostasis (Cannon, 1929). Research shows that non-linear coupling between integrated physiological systems such as the cardiac, respiratory, and cardiovascular systems exhibits complex dynamic characteristics and is regulated by internal feedback mechanisms (Dick et al., 2014). The mechanical stretch caused by respiratory movements and the resulting changes in cardiac output cause the periodic fluctuation of blood pressure. Blood pressure signals received by baroreceptors are transmitted to the central nervous system via the baroreflex pathway; signals from the central nervous system then regulate systemic vascular resistance as well as the electrical and mechanical activity of the heart (via the sympathetic and vagal neural pathways of the autonomic nervous system, respectively) to achieve feedback regulation of cardiac output (Garcia et al., 2013; Schulz et al., 2013; Dick et al., 2014; Ottolina et al., 2023). Complex functional and anatomical coupling between cardiac, respiratory, and blood pressure activities is achieved through highly overlapping brainstem networks to regulate autonomic physiological processes that are critical to survival. However, previous studies on the immediate effects of EECP focused only on hemodynamics or cardiovascular function, ignoring the potential role of physiological coupling between the cardiac, respiratory, and cardiovascular systems.

To test the hypothesis that physiological coupling quantified by phase synchronization index (PSI) is altered by immediate EECP intervention, using simultaneous multi-modal physiological recordings, we assessed PSI, HRV measurements, and hemodynamic parameters in the active EECP and sham control subjects before, during and after EECP. To answer the question of whether presumed alterations of PSI are related to the hemodynamic characteristics *per se* or associated cardiovascular changes, we additionally performed regression analysis between PSI and hemodynamic measurements.

## 2. Materials and methods

### 2.1. Study subjects

This study was conducted in the Hyperbaric Oxygen Department of the Chinese PLA General Hospital. From July 8, 2021, to September 10, 2021, healthy subjects from Beijing Sport University and Chinese PLA Medical School were recruited through advertisements. After excluding female subjects due to significant variations in vascular function during the phases of the menstrual cycle, individuals with various acute and chronic diseases, drug use, alcohol/caffeine addiction, smoking, and other factors that may have affected the autonomic function and test results, 46 healthy subjects were included. The subjects were randomly divided into an EECP group (23 people) and a control group (23 people). This study was approved by the Medical Ethics Committee of the Chinese PLA General Hospital (No. S2019-204-01). All healthy subjects were informed about the study and signed an informed consent form before the trial. The research project was registered with the Chinese Clinical Trial Registry (No. ChiCTR2000033645).

### 2.2. Data collection

Healthy subjects were randomly assigned (1:1) to either intervention with active EECP stimulation (EECP group) or intervention with sham EECP stimulation (control group) using a simple computer-generated sequence. Participants from the EECP and control groups participated in the trial in the EECP treatment room, which was kept at 22–25^°^C. To eliminate or reduce the impact of circadian rhythms on the physiological systems under examination, the trials were completed between 9:30 a.m. and 11:00 a.m. All subjects were in a supine position throughout the trial. The 10 min period after 5 min of rest was defined as the stage before the EECP interventions (Baseline). The next stage was the EECP intervention stage, during which the EECP group received two consecutive EECP interventions (Chongqing PSK-Health Sci-Tech Development Co., Ltd., P-ECP/TI, Chongqing, China) with a pressure parameter of 0.020 Mpa and duration of 30 min (with a 5 min interval between EECP-1 and EECP-2). The control group went through the same process as the EECP group, except that the pressure parameter was set to 0 Mpa. The subsequent 10 min period was defined as the stage after the end of the EECP intervention (Post-EECP). Multimodal physiological signals and hemodynamic parameters were recorded and saved throughout the trial.

This study used the Biopac MP160 multi-channel physiological recording system (Biopac Systems Inc., Goleta, CA, USA) to simultaneously acquire multi-modal physiological signals such as ECG (using standard limb lead II configuration), blood oxygen saturation, photoplethysmography (PPG), and chest bioelectrical impedance. The signal sampling frequency was 2,000 Hz. The storage format was “. mat” to facilitate subsequent analysis and processing based on MATLAB. All volunteers wore a pressure-measuring cuff on their left upper arm and had fingertip pressure sensors placed on their right index fingers and middle fingers. Hemodynamic data were recorded through the CNAP system 500 (CNSystems Medizintechnik GmbH, Graz, Austria) at a sampling frequency of 100 Hz. Subsequently, the collected hemodynamic data were segmented according to the stages before, during (two 30 min EECP interventions), and after the EECP interventions. The average values for each segment were calculated to obtain characteristic parameters such as systolic blood pressure (SBP), diastolic blood pressure (DBP), mean arterial pressure (MAP), cardiac output (CO), cardiac index (CI), stroke volume variability (SVV), and systemic vascular resistance (SVR).

**FIGURE 1:**
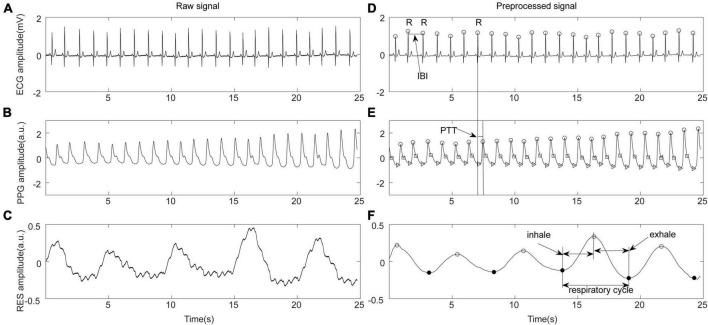
Schematic diagram of physiological signal preprocessing. **(A)** Original electrocardiograph (ECG) signal. **(B)** Original photoplethysmography (PPG) signal. **(C)** Original respiration (RES) signal. **(D)** ECG preprocessing, R wave recognition, and definition of inter-beat interval (IBI). **(E)** PPG feature point recognition and pulse transit time (PTT) calculation. **(F)** Respiratory signal preprocessing and feature extraction.

Multimodal physiological signals and hemodynamic data were preprocessed using a 50 Hz notch filter in MATLAB R2020 (MathWorks, Natick, MA, USA) to eliminate power frequency interference. As shown in Figure 1, after band-pass filtering at 0.5–40 Hz, the peak points of the ECG R wave and PPG wave were detected using Kubios software (Kubios 2.2, University of Eastern Finland, Kuopio). Based on the detected R-wave feature points, the interbeat interval (IBI) sequence was constructed. After manual inspection, abnormal points with IBI > 2,000 ms, IBI < 300 ms, adjacent IBI changes > 200 ms, and IBI change with ranges exceeding 20% of the mean value of the previous five IBIs were eliminated (Liu et al., 2020). The time differences between the peak point of the ECG R wave and the peak point of the PPG in the same cycle were calculated to construct the pulse transit time (PTT) sequence and characterize the beat-by-beat blood pressure (Hoshide et al., 2022). The respiration (RES) signal was extracted from the chest bioelectrical impedance signal via a 0.05–0.8 Hz band-pass filter and the respiratory rate (RR) was calculated. The preprocessed IBI, PTT, and RES time series were resampled at 5 Hz for further analysis.

### 2.3. Analysis of physiological coupling

**FIGURE 2:**
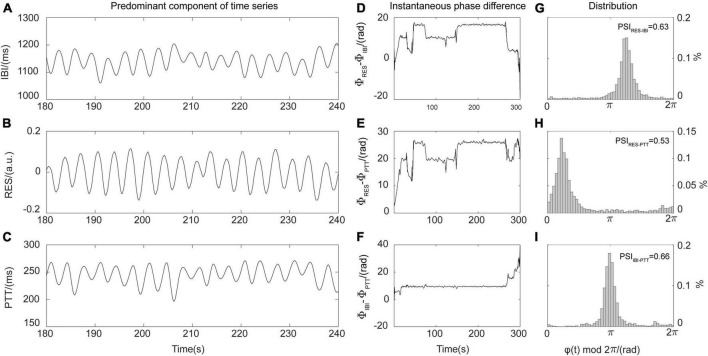
Schematic diagram of phase synchronization index (PSI) calculations. **(A)** Predominant components of the interbeat interval (IBI) time series. **(B)** Predominant components of the respiration (RES) signals. **(C)** Predominant components of the pulse transit time (PTT) series. **(D)** Instantaneous phase difference between RES signal and IBI time series (φ_RES–IBI_). **(E)** Instantaneous phase difference between RES signal and PTT time series (φ_RES–PTT_). **(F)** Instantaneous phase difference between IBI and PTT time series (φ_IBI–PTT_). **(G)** Distribution diagram for instantaneous phase difference φ_RES–IBI_ and PSI_RES–IBI_. **(H)** Distribution diagram for instantaneous phase difference φ_RES–PTT_ and PSI_RES–PTT_. **(I)** Distribution diagram for instantaneous phase difference φ_IBI–PTT_ and PSI_IBI–PTT_.

Phase synchronization refers to the synchronization of the oscillations of two mutually coupled systems; that is, the phase difference between the two systems is fixed and does not change with time. The PSI is often used to quantify the strength of coupling between different physiological systems, especially the respiratory and cardiovascular systems (Zheng et al., 2016; Mejía-Mejía et al., 2018). This study divided the RES, IBI, and PTT time series resampled during the stages before, during, and after the EECP interventions into segments of equal length (1 min). Each segment was initially processed using empirical mode decomposition (EMD) and was expressed as


(1) $$x\,\left(t\right)=\sum_{i=1}^{n}c_{i}\,\left(t\right)+r_{n}\,\left(t\right)$$

where *c*_*i*_(*t*) is the intrinsic mode function, and *r*_*n*_(*t*) is the residual. At a resampling frequency of 5 Hz, the No. *m* intrinsic mode function *c*_*m*_(*t*) of the RES, IBI, and PTT time series was selected as the main component of the above three physiological signal time sequences, as shown in Figure 2. On this basis, the Hilbert transform pair *c*_*m*_(*t*) of ${\hat{c}}_{m}\,\left(t\right)$ was constructed using the Hilbert transform:


(2) $${\hat{c}}_{m}\,\left(t\right)=\frac{1}{\pi }\,p.v.\int_{-\infty }^{+\infty }\frac{c_{m}\,\left(\tau \right)}{t-\tau }\,d\tau$$

Here, *p*.*v*. is the Cauchy principal value, while the analytic signal *z*_*m*_(*t*) is defined by the complex conjugate transformation pair *c*_*m*_(*t*) and ${\hat{c}}_{m}\,\left(t\right)$. Its analytical form is


(3) $$z_{m}\,\left(t\right)=c_{m}\,\left(t\right)+{\hat{c}}_{m}\,\left(t\right)\equiv A\,\left(t\right)\,e^{j\,\Phi \,t}$$

where $A\,\left(t\right)=\sqrt{c_{m}\,{\left(t\right)}^{2}+{\hat{c}}_{m}\,{\left(t\right)}^{2}}$, $\Phi \,\left(t\right)=\arg\,\tan\,\frac{{\hat{c}}_{m}\,\left(t\right)}{c_{m}\,\left(t\right)}$. In this way, the Hilbert-Huang transform provides the instantaneous phase Φ(*t*) of *c*_*m*_(*t*). The instantaneous phases of the main components of synchronous RES, IBI, and PTT time series are, respectively recorded as Φ_*RES*_(*t*), Φ_*IBI*_(*t*), and Φ_*PTT*_(*t*). Then, it is possible to determine the instantaneous phase difference φ(*t*) between RES, IBI, and PTT, namely, φ_*RES*−*IBI*_(*t*)=Φ_*RES*_(*t*)−Φ_*IBI*_(*t*), φ_*RES*−*PTT*_(*t*)=Φ_*RES*_(*t*)−Φ_*PTT*_(*t*), and φ_*IBI*−*PTT*_(*t*)=Φ_*IBI*_(*t*)−Φ_*PTT*_(*t*). The PSI representing the strength of coupling between respiratory, cardiac, and blood pressure systems is calculated as


(4) $$P\,S\,I={\left(\bar{c\,o\,s\,\varphi \,\left(t\right)}\right)}^{2}+{\left(\bar{s\,i\,n\,\varphi \,\left(t\right)}\right)}^{2}$$

where $\bar{c\,o\,s\,\varphi \,\left(t\right)}$ and $\bar{s\,i\,n\,\varphi \,\left(t\right)}$ represent the means of the cosine and sine values of the instantaneous phase difference φ(**t**) between two 1 min time series. In theory, 0≤*PSI*≤1; for the oscillation time series of two real-world systems, when the instantaneous phase difference is constant (that is, PSI = 1), the two systems are fully synchronized. In contrast, at PSI values <0.14, the two systems can be considered to be completely out of synchronization (Cysarz and Büssing, 2005). By averaging all the PSI segments of equal length before, during, and after the EECP interventions, it was possible to obtain the values *PSI*_*RES–IBI*_, *PSI*_*RES–PTT*_, and *PSI*_*IBI–PTT*_, which quantified the strength of coupling between respiration and heart, respiration and blood pressure, and heart and blood pressure systems, respectively. As shown in Figure 2, the higher the degree of coupling between the oscillations of two systems, the stronger the synchronization, the larger the corresponding PSI value, and the more obvious the peak value displayed in the distribution diagram of instantaneous phase difference. To characterize the total strength of physiological coupling, we defined the index *PSI*_*total*_ to represent the interactions between respiratory, cardiac, and cardiovascular systems (Ocon et al., 2011; Liu et al., 2022):


(5) $$P\,S\,I_{t\,o\,t\,a\,l}=P\,S\,I_{R\,E\,S-I\,B\,I}+P\,S\,I_{R\,E\,S-P\,T\,T}+P\,S\,I_{I\,B\,I-P\,T\,T}$$

### 2.4. Heart rate variability analysis

Heart rate variability time-domain measures included the mean heart-beat intervals (Mean IBI), standard deviation of the IBIs (SDNN), and square root of the mean of sum of squares of the differences between adjacent IBIs (RMSSD) were calculated from the 10 min ECG recordings using recommended methods. Fast Fourier transform was used to quantify the main spectral components for the total power (TP) for the frequency range 0.0033–0.40 Hz; the low-frequency power (LF) for the frequency range 0.04–0.15 Hz and the high-frequency power (HF) for the frequency range 0.15–0.40 Hz and the ratio of LF to HF (LF/HF) (Malik et al., 1996). The non-linear measure of approximate entropy (ApEn) was also calculated to quantify the complexity or regularity of the HRV time series by measuring the unpredictability of fluctuation patterns (Pincus, 1991).

### 2.5. Statistical analysis

Continuous variables such as physiological coupling indexes, hemodynamic parameters, physiological measurements, demographic characteristics, and laboratory test data were expressed as mean ± standard deviation. For dichotomous or categorical variables, Fisher’s exact test was used for statistical analysis, and the Mann-Whitney U test was used to compare differences in baseline clinical characteristics between the EECP and control groups. Intra-group and inter-group differences in physiological coupling indexes, hemodynamic parameters and physiological measurements in the EECP and control groups before, during, and after the EECP intervention were tested by two-way repeated measures ANOVA with Bonferroni post hoc correction. A multiple linear regression model was used to analyze the relationship between the changes in the physiological coupling indexes and the changes in the hemodynamic parameters following EECP. All statistical analyses were performed using software SPSS 25.0 (SPSS, Chicago, IL, USA); *P* < 0.05 indicated a statistically significant difference.

## 3. Results

### 3.1. Clinical data results

Preliminary analysis of the data showed that the physiological data for five subjects were missing (one in the EECP group and four in the control group). Therefore, a total of 41 healthy subjects (24 males and 17 females) with a mean age of 23.1 ± 2.3 years and a mean body mass index of 22.08 ± 2.49 kg/m^2^ were finally included in this study. Table 1 shows the demographic characteristics, physiological indicators, and clinical blood test results for the healthy subjects in the EECP and control groups. There were no statistically significant differences between the two groups in terms of gender composition, age, blood pressure, heart rate, RR, oxygen saturation, or routine blood and biochemical parameters (*P >* .05).

**TABLE 1:** Demographic characteristics and clinical indicators of the study population.

Parameters	EECP group (*n* = 22)	Control group (*n* = 19)	*P*-value
Gender, *n* (%)	–	–	0.113
Male	12 (54.5)	12 (63.2)	–
Female	10 (45.5)	7 (36.8)	–
Age (years)	22.6 ± 2.1	23.6 ± 2.5	0.130
Height (cm)	171 ± 8	173 ± 11	0.380
Weight (kg)	63.6 ± 10.9	68.6 ± 13.3	0.260
BMI (kg/m^2^)	21.64 ± 2.67	22.59 ± 2.21	0.302
Systolic pressure (mmHg)	106 ± 15	110 ± 10	0.346
Diastolic pressure (mmHg)	68 ± 14	64 ± 8	0.142
Mean blood pressure (mmHg)	84 ± 12	82 ± 8	0.366
Heart rate (bpm)	64 ± 12	67 ± 10	0.224
RR (rpm)	18.3 ± 2.3	18.9 ± 3.3	0.721
Oxyhemoglobin saturation (%)	97 ± 1	96 ± 2	0.064
Hemoglobin (g/L)	139 ± 13	144 ± 13	0.308
Erythrocyte (10^12^/L)	4.65 ± 0.44	4.71 ± 0.53	0.855
Leukocyte (10^9^/L)	6.68 ± 1.36	6.09 ± 1.37	0.234
Thrombocyte (10^9^/L)	225 ± 59	237 ± 54	0.601
Glucose (mmol/L)	5.42 ± 0.90	5.12 ± 0.71	0.539
Uric acid (μmol/L)	330.8 ± 75.4	344.0 ± 94.2	0.937
Cholesterol (μmol/L)	4.10 ± 0.76	3.92 ± 0.66	0.556
Creatine kinase (mmol/L)	126.5 ± 78.7	139.9 ± 116.1	0.472
Potassium ion concentration (mmol/L)	4.12 ± 0.19	4.11 ± 0.28	0.804
Cystatin C (mg/L)	0.77 ± 0.10	0.73 ± 0.13	0.234

### 3.2. Immediate physiological coupling effect of EECP

**FIGURE 3:**
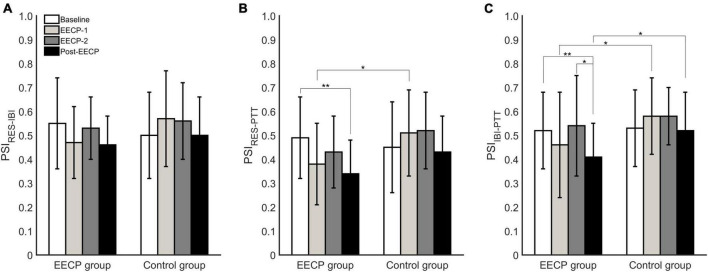
Analysis of phase synchronization index changes in respiration (RES), interbeat interval (IBI), and pulse transit time (PTT) before, during, and after the two 30 min enhanced external counterpulsation (EECP) interventions in the EECP and control groups. **(A)** PSI_RES–IBI_ between RES and IBI time series. **(B)** PSI_RES–PTT_ between RES signal and PTT series. **(C)** PSI_IBI–PTT_ between IBI time series and PTT series. Statistical significance was determined by two-way repeated measures ANOVA; *P < 0.05, **P < 0.01.

As shown in Figure 3, analysis of the coupling indexes indicated that the cardiorespiratory coupling parameter PSI_RES–IBI_ was not significantly different in the EECP and control groups before, during or after the EECP intervention (all *P >* 0.05). However, in the EECP group, the respiratory–blood pressure coupling index PSI_RES–PTT_ in Post-EECP stage was significantly lower than that of Baseline (0.34 ± 0.14 vs. 0.49 ± 0.17, *P* = 0.002). In addition, the PSI_RES–PTT_ of the EECP group was significantly lower than that of the control group during EECP-1 (0.38 ± 0.17 vs. 0.51 ± 0.18, *P* = 0.02). Similarly, the cardiovascular coupling index PSI_IBI–PTT_ was significantly lower in the EECP group in Post-EECP, compared with Baseline (0.41 ± 0.14 vs. 0.52 ± 0.16, *P* = 0.006) and during EECP-2 (0.41 ± 0.14 vs. 0.54 ± 0.21, *P* = 0.02). The PSI_IBI–PTT_ values of the EECP group were also significantly lower than the levels observed for the control group during EECP-1 (0.46 ± 0.21 vs. 0.58 ± 0.16, *P* = 0.05) and Post-EECP (0.41 ± 0.14 vs. 0.52 ± 0.16, *P* = 0.03).

**FIGURE 4:**
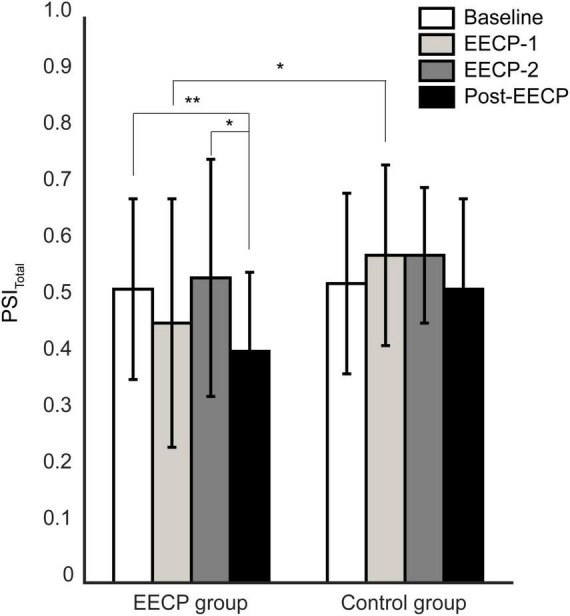
Analysis of changes in total coupling strength between respiratory and cardiovascular systems before, during, and after the two 30 min enhanced external counterpulsation (EECP) interventions in the EECP and control groups. Statistical significance was determined by two-way repeated measures ANOVA; *P < 0.05, **P < 0.01.

As shown in Figure 4, the PSI_total_ changes showed a similar trend to the changes in PSI_IBI–PTT_ values. In the EECP group, the total coupling intensity PSI_total_ between respiratory and cardiovascular systems was significantly lower at Post-EECP than those at baseline (1.21 ± 0.35 vs. 1.57 ± 0.49, *P* = 0.005) and during EECP-2 (1.21 ± 0.35 vs. 1.50 ± 0.42, *P* = 0.03). In addition, the PSI_total_ was significantly lower in the EECP group than that in the control group during EECP-1 (1.30 ± 0.46 vs. 1.66 ± 0.51, *P* = 0.02).

### 3.3. Immediate hemodynamic and physiological effects of EECP

As shown in Table 2, in the EECP group, SBP (96 ± 11 vs. 105 ± 19, *P* = 0.02), DBP (54 ± 7 vs. 61 ± 14, *P* = 0.04), and SVR (850 ± 285 vs. 1027 ± 330, *P* = 0.008) were significantly reduced during EECP-1, compared with the Baseline. In addition, CO (7.68 ± 2.85 vs. 6.32 ± 1.28, *P* = 0.02) and CI (4.57 ± 2.03 vs. 3.73 ± 0.99, *P* = 0.02) were significantly increased, compared with the Baseline period. Meanwhile, SVV was significantly altered during EECP-1 (18.26 ± 7.16 vs. 11.97 ± 3.47, *P <* 0.001) and EECP-2 (20.29 ± 7.42 vs. 11.97 ± 3.47, *P <* 0.001). In contrast, there were no statistically significant differences in blood pressure, CO, CI, or SVR during or after EECP intervention, compared with Baseline. In the control group, no significant changes in hemodynamic parameters were observed during EECP-1, EECP-2, or the Post-EECP period, compared with those before EECP intervention. The EECP group showed that during EECP-1, CO (5.98 ± 1.71 vs. 7.68 ± 2.85, *P* = 0.03), CI (3.36 ± 0.86 vs. 4.57 ± 2.03, *P* = 0.02) and SVV (11.78 ± 3.75 vs. 18.26 ± 7.16, *P* = 0.001) were significantly lower in the control group, while SVR was significantly higher (1072 ± 295 vs. 850 ± 285, *P* = 0.02). In addition, during EECP-2, SVV was significantly lower in the control group, compared with that in the EECP group (11.37 ± 4.47 vs. 20.29 ± 7.42, *P <* 0.001).

**TABLE 2:** Hemodynamic parameters before, during, and after the two 30 min enhanced external counterpulsation (EECP) interventions in the enhanced external counterpulsation (EECP) and control groups.

Indicators	EECP group (*n* = 22)				Control group (*n* = 19)			
	Baseline	EECP-1	EECP-2	Post-EECP	Baseline	EECP-1	EECP-2	Post-EECP
Systolic pressure (mmHg)	105 @ 19	96 @ 11*	99 @ 12	103 @ 16	106 @ 13	102 @ 11	104 @ 15	102 @ 15
Diastolic pressure (mmHg)	61 @ 14	54 @ 7*	55 @ 9	59 @ 13	64 @ 11	59 @ 11	56 @ 11	57 @ 12
Mean blood pressure (mmHg)	79 @ 14	73 @ 7	76 @ 10	77 @ 12	81 @ 10	75 @ 9	72 @ 10	74 @ 11
Cardiac output (Lmin^–1^)	6.32 @ 1.28	7.68 @ 2.85*	7.17 @ 2.41	6.37 @ 1.64	6.12 @ 0.73	5.98 @ 1.71^#	6.15 @ 1.64	5.99 @ 1.21
Cardiac index (Lmin^–1^m^–2^)	3.73 @ 0.99	4.57 @ 2.03*	4.24 @ 1.60	4.04 @ 1.97	3.47 @ 0.54	3.36 @ 0.86^#	3.44 @ 0.80	3.38 @ 0.66
Stroke volume variation (%)	11.97 @ 3.47	18.26 @ 7.16***	20.29 @ 7.42***	11.76 @ 4.04	11.57 @ 4.59	11.78 @ 3.75^##	11.37 @ 4.47^###	10.96 @ 3.94
Systemic vascular resistance (dynscm^–5^)	1,027 @ 330	850 @ 285*	934 @ 345	1,006 @ 333	1,095 @ 228	1,072 @ 295^#	993 @ 244	1,018 @ 283

Statistical significance of hemodynamic parameters during and after the two enhanced external counterpulsation (EECP) interventions, compared with those of baseline: * and *** indicate *P* < 0.05 and *P* < 0.001, respectively; statistical significance of comparison of hemodynamic parameters in the EECP and control groups: ^#^, ^##^, and ^###^ indicate *P* < 0.05, *P* < 0.01, and *P* < 0.001, respectively.

The results of HRV, PTT and RR analyses in the EECP and control groups are presented in Table 3. In the EECP group, two sessions of EECP significantly increased SDNN compared to that of Baseline (99 ± 37 vs. 71 ± 25, *P <* 0.001). PTT for both stages of EECP-1 (341 ± 18 vs. 334 ± 20, *P* = 0.012) and EECP-2 (346 ± 19 vs. 334 ± 20, *P <* 0.001) were significantly increased compared to those before the EECP intervention. In the control group, both SDNN and Mean IBI for EECP-1, EECP-2, and Post-EECP were significantly higher than those of stage Baseline (all *P <* 0.05). In addition, the RMSSD during EECP-1 (76 ± 45 vs. 61 ± 39, *P* = 0.005) and EECP-2 (75 ± 41 vs. 61 ± 39, *P* = 0.022) periods were significantly increased compared with those before intervention. No significant changes in frequency-domain and non-linear measurements were found in both the EECP and control groups. Furthermore, there were no significant differences in all the analyzed HRV indices, PTT and RR between the EECP and control groups.

**TABLE 3:** Mean ± standard deviation of heart rate variability (HRV) measurements, pulse transit time (PTT), and respiratory rate (RR) before, during, and after the two 30 min enhanced external counterpulsation (EECP) interventions in the enhanced external counterpulsation (EECP) and control groups.

Indicators	EECP group (*n* = 22)				Control group (*n* = 19)			
	Baseline	EECP-1	EECP-2	Post-EECP	Baseline	EECP-1	EECP-2	gPost-EECP
SDNN (ms)	71 @ 25	73 @ 27	81 @ 32	99 @ 37***	67 @ 28	81 @ 31*	88 @ 31**	92 @ 29***
RMSSD (ms)	60 @ 28	57 @ 27	65 @ 32	60 @ 24	61 @ 39	76 @ 45**	75 @ 41*	70 @ 34
LF (ms^2^)	1,154 @ 1,019	1,193 @ 962	1,431 @ 1,503	1,558 @ 946	1,172 @ 1,139	1,105 @ 943	1,283 @ 1013	1,481 @ 881
HF (ms^2^)	1,307 @ 1075	1,199 @ 1,105	1,515 @ 1,277	1,275 @ 881	1,598 @ 2,167	2,251 @ 3,164	2,217 @ 2,767	1,939 @ 1,875
TP (ms^2^)	2,615 @ 2,046	2,512 @ 1,872	3,090 @ 2,615	3,074 @ 1,785	2,908 @ 3,226	3,508 @ 3,855	3,693 @ 3,517	3,603 @ 2,246
LF/HF	1.11 @ 1.02	1.32 @ 1.00	1.22 @ 1.07	1.48 @ 0.86	1.34 @ 1.48	0.83 @ 0.75	0.97 @ 0.82	1.41 @ 1.34
ApEn	1.26 @ 0.11	1.24 @ 0.10	1.21 @ 0.09	1.25 @ 0.10	1.22 @ 0.09	1.21 @ 0.06	1.19 @ 0.06	1.22 @ 0.06
Mean IBI (ms)	985 @ 152	1,009 @ 181	1,033 @ 167	985 @ 150	995 @ 184	1,092 @ 173***	1,106 @ 171***	1,076 @ 182**
PTT (ms)	334 @ 20	341 @ 18*	346 @ 19***	338 @ 17	330 @ 18	335 @ 20	336 @ 19	337 @ 19
RR (rpm)	18.3 @ 1.8	19.2 @ 3.3	19.3 @ 3.0	17.9 @ 2.4	19.0 @ 3.5	18.7 @ 2.5	17.8 @ 2.2	17.8 @ 2.1

Statistical significance of heart rate variability (HRV) parameters, pulse transit time (PTT), and respiratory rate (RR) during and after the two enhanced external counterpulsation (EECP) interventions, compared with those of baseline: *, **, and *** indicate *P* < 0.05, *P* < 0.01, and *P* < 0.001, respectively.

### 3.4. Regression analysis

**FIGURE 5:**
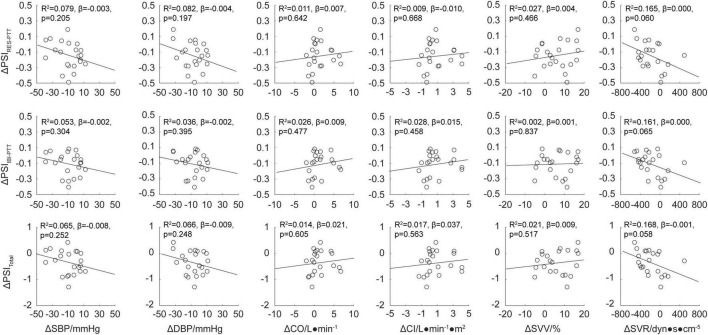
Regression analysis for the physiological coupling indexes ΔPSI_RES–PTT_, ΔPSI_IBI–PTT_, and ΔPSI_total_ and hemodynamic parameters ΔSBP, ΔDBP, ΔCO, ΔCI, ΔSVV, and ΔSVR after enhanced external counterpulsation (EECP) intervention. The line of best fit was used to characterize the linear correlation between two parameters; values for R^2^, β, and p are displayed.

Multiple he reduced PSI indluinear regression analysis was used to study the relationship between the changes in the physiological coupling indexes, ΔPSI_RES–PTT_, ΔPSI_IBI–PTT_, and ΔPSI_total_ (which were significantly changed from baseline values after the EECP intervention), and the changes in the hemodynamic parameters, ΔSBP, ΔDBP, ΔCO, ΔCI, ΔSVV, and Δ SVR. The results are shown in Figure 5. There were no significant correlations between the immediate effects of physiological coupling induced by EECP and the immediate hemodynamic effects (all *P* > 0.05).

## 4. Discussion

In this study, we aimed to investigate the immediate physiological coupling effects of EECP by means of phase synchronization analysis. The results showed that during EECP, hemodynamic parameters were significantly altered. However, the strength of coupling between heartbeat, respiration, and blood pressure, and the total coupling strengths of the integrated cardiorespiratory and cardiovascular physiological systems were not significantly affected. In contrast, after EECP, the physiological coupling indexes PSI_RES–PTT_, PSI_IBI–PTT_, and PSI_total_ were significantly reduced. However, there was no significant correlation between the reduction in the physiological coupling indexes and the changes in hemodynamic parameters.

The coupling of the integrated cardiorespiratory and cardiovascular physiological systems reflects the interaction of the autonomic nervous system and respiratory control system (Fisher et al., 2022). Such interactions involve not only the influence of respiration on heart rate and blood pressure but also the regulation of respiration and ventilation patterns by the cardiovascular system. HRV analysis is often used as a non-invasive and effective tool for the assessment of autonomic function (Siepmann et al., 2022; Grégoire et al., 2023). In the present study, frequency-domain measures LF (reflects both sympathetic and parasympathetic control of the heart rate), HF (generally interpreted as a marker of vagal activity and is respiration mediated) and LF/HF (reflects the global sympathovagal balance) were not altered by EECP. Similarly, spontaneous RR, which is closely related to autonomic function, has not undergone significant changes during and after EECP for both groups. However, breathing patterns with inter-and/or intra-individual variability would probably mask the effects of EECP on physiological coupling and cardiovascular autonomic function. The synergistic effects resulting from the coupling of physiological systems are essential for the maintenance and promotion of a healthy state in the human body (Garcia et al., 2013; Dick et al., 2014). In the field of medical research into complex cardiorespiratory and cardiovascular systems, increasing attention is being paid to the interaction between changes in physiological coupling states and their regulatory mechanisms in healthy individuals and patients with different diseases. Preliminary analyses of diagnosis, risk stratification, outcome prediction, and motor function assessment in conditions such as COPD, sleep apnea, type 2 diabetes mellitus, depression, and premature infant birth have shown that disease states are often accompanied by dysregulated physiological coupling (Lucchini et al., 2018; Zhao et al., 2019; Huang et al., 2021; Abreu et al., 2022; Silva et al., 2023). Dysregulation, generally taking the form of reduced physiological coupling strength, involves several complex biological mechanisms and may be related to the autonomic regulation of the cardiorespiratory and cardiovascular systems.

Studies have shown that EECP has complex immediate hemodynamic effects, resulting in increased aortic diastolic pressure, decreased systolic pressure, increased cardiac output, and increased coronary and cerebral blood flow (Li et al., 2018; Lin et al., 2020), these changes may be the basis of the rehabilitative effects of EECP in cardiovascular diseases. In addition, EECP treatment of coronary artery disease and angina is accompanied by long-term effects such as increased HRV and improved cardiac autonomic function (Xiong et al., 2017, 2020; Ziad et al., 2020). With regard to the immediate effect of EECP on HRV, significant changes in beat-to-beat HRV were not observed during ischemic stroke, while the HRV frequency domain indicators LF and TP increased significantly after EECP (Xiong et al., 2017). Although the cardiorespiratory and cardiovascular systems are complex, non-linear, and integrated coupled physiological systems, current studies on the physiological effects of EECP have focused only on local indicators such as HRV or hemodynamics, neglecting its overall effects. This study confirmed the changes in hemodynamic properties during EECP in a healthy population and further revealed the immediate effects of EECP in terms of the reduced coupling strength of the integrated cardiorespiratory and cardiovascular physiological systems. This is consistent with previous findings regarding the use of EECP by athletes to suppress the vagal activity of the autonomic nervous system during recovery from exercise fatigue (Valenzuela et al., 2018). Similarly, negative lower body pressure interventions increased the intensity of heartbeat–blood pressure coupling in the spontaneous state (Negulyaev et al., 2021).

In this study, there were two sessions of EECP interventions. The main hemodynamic parameters such as SBP, DBP, CO, CI, and SVR were changed during the first EECP. PTT, which is inversely related to blood pressure (Block et al., 2020), was increased significantly by EECP intervention. The result is partially consistent with the findings of EECP on blood pressure (Tian et al., 2021; Zhang et al., 2021). At the same time, the PSI_RES–IBI_, PSI_RES–PTT_, PSI_IBI–PTT_, and PSI_total_ indicators, which characterize the coupling strength of the integrated cardiorespiratory and cardiovascular physiological systems, were decreased. The corresponding hemodynamic and physiological coupling indexes were essentially the same as baseline values during the second EECP intervention, indicating that EECP may induce a “saturation” effect. That is, EECP interventions beyond a specific time may cause changes in the stability of the integrated cardiorespiratory and cardiovascular physiological systems, leading to their inability to respond effectively to the input. This finding has implications for the optimization of EECP techniques and therapies. In addition, the immediate physiological coupling effects of EECP were significantly delayed, compared with the hemodynamic effects, meaning that the reduction of PSI_RES–PTT_, PSI_IBI–PTT_, and PSI_total_ indicators occurred after, rather than during EECP. This may be a compensatory response by the integrated cardiorespiratory and cardiovascular physiological systems to the EECP-induced redistribution of blood flow and its recovery process. Although EECP reduced the intensity of physiological coupling in healthy subjects, the immediate physiological coupling effects of EECP in patients and the relationship between these effects and therapeutic efficacy remain unclear. Therefore, in the field of EECP treatment of ischemic cardiovascular diseases, it is crucial to investigate the mechanism of physiological coupling and continuously optimize intervention strategies.

There are several limitations in the present study. First, the hemodynamic data collected in our study is relatively limited, and there is no acquisition of cerebral blood flow and multi-channel electroencephalography signals. So it is unknown whether EECP has an immediate effect on cerebrovascular coupling or brain-heart coupling. Second, we investigated the immediate effects of EECP on healthy volunteers. The sustained or long-term effects of EECP intervention on physiological coupling and their relation to clinical efficacy were not determined. Third, only one of the coupling indices PSI was analyzed, other physiological coupling measures such as magnitude squared coherence and phase to amplitude estimator that may extend the analysis were not covered. Further studies performed with standardized protocols on a larger scale in risky cohorts are warranted.

## 5. Conclusion

For the first time, this study reveals the immediate physiological coupling effects of EECP using phase synchronization analysis. The findings complement our understanding of the physiological and hemodynamic effects of EECP and provide new perspectives and clues for exploring the mechanism of action of EECP. The study results will not only aid in the optimization of EECP technology and therapy but could also be applied clinically to predict the efficacy of EECP treatment in ischemic cardiovascular diseases.

## Data availability statement

The original contributions presented in this study are included in the article/supplementary material, further inquiries can be directed to the corresponding authors.

## Ethics statement

The studies involving human participants were reviewed and approved by the research project was registered with the Chinese Clinical Trial Registry (No. ChiCTR2000033645). The patients/participants provided their written informed consent to participate in this study.

## Author contributions

HLiu and HLia: conceptualization, writing—original draft preparation, and formal analysis. XY, YH, and GW: data curation and visualization. MY and WW: supervision and writing—review and editing. SL: writing—review and editing and funding acquisition. All authors contributed to the article and approved the submitted version.
